# Use of a Survey to Assess the Environmental Exposure and Family Perception to Lead in Children (<6 Years) in Four Valley Cities, Northwestern China

**DOI:** 10.3390/ijerph15040740

**Published:** 2018-04-12

**Authors:** Xuemeng Sun, Xiaoping Li, Dongying Liu, Tao Yang, Yanan Zhao, Ting Wu, Yue Cai, Yuwei Ai, Xu Zhang, Jiwen Wang, Rui Yang, Hongtao Yu, Howard W. Mielke

**Affiliations:** 1 Department of Environmental Science, School of Geography and Tourism, Shaanxi Normal University, Xi’an 710062, China; sunxuemeng@snnu.edu.cn (X.S.); liu_dongying@yeah.net (D.L.); yangfan2288@163.com (T.Y.); zhaoyanan@snnu.edu.cn (Y.Z.); lanmiao0913@snnu.edu.cn (T.W.); caiyue@snnu.edu.cn (Y.C.); aiyuwei@snnu.edu.cn (Y.A.); zhangxu_00@snnu.edu.cn (X.Z.); kc2301wangjiwen@snnu.edu.cn (J.W.); yangrui1258@163.com (R.Y.); 2 International Joint Research Centre of Shaanxi Province for Pollutant Exposure and Eco-Environmental Health, Xi’an 710062, China; hongtao.yu@morgan.edu; 3 School of Computer, Mathematical and Natural Sciences, Morgan State University, Baltimore, MD 21251, USA; 4 Department of Pharmacology, Environmental Signaling Laboratory, Tulane University School of Medicine, 1430 Tulane Avenue SL-8683, New Orleans, LA 70112, USA; hmielke@tulane.edu

**Keywords:** lead exposures, children’s blood lead, valley cites, family, behaviors and habits, parent perception

## Abstract

With the growth of industry, the extensive use of lead, and urban expansion in Northwestern Valley Cities (NVC) China, there is probable reason for presuming an increasing risk of lead exposure. However, little is known about the lead exposure of children less than 6 years old in NVC. As a first investigation, this study uses a survey to systematically determine the influences of various risk factors within the family environment, parents’ background, children’s behavior, mother’s behavior during pregnancy, and parental perception about children’s blood lead (CBL). A total of 596 families were recruited from the general population in Urumqi, Lanzhou, Xining and Yan’an. Parents, and their children (<6 years old), were asked about the environment and behaviors which could possibly relate with lead exposure. The results indicated that in the typical NVC of China, children’s environment and behavior, parents’ education level, and mother’s pregnancy behavior, were associated with potential CBL. It was noted that not all parents in NVC China recognized the importance of children’s lead exposure. Therefore, children’s health care and medical screening campaigns need to be designed to improve family’s fundamental knowledge of lead hazards, associated health effects, and prevention in the NVC of China.

## 1. Introduction

### 1.1. Lead Pollution

The use of lead and its compounds has a long history dating back to Roman Empire, which was the first peak of global lead production [1]. During the Roman period, lead was used to manufacture water aqueducts supplying the urban domestic water and people only saw the convenience of the lead pipes at that time. With the development of the urbanization and industrialization, lead has been widely used in various aspects of production and life, relying on its own characteristics which cannot be fully replaced by other materials [2,3]. At present, lead consumption in the world mainly focuses on lead–acid batteries [4], leaded water pipes [5], paints pigments [6,7], and alloys and ammunition [7]. Lead is found in nature mainly as a sulfide mineral, not as a metal. Therefore, anthropogenic smelting activities are the main reason for the wide spread occurrence of lead in the environment.

The many ways lead is used results in a variety of potential exposure sources and pathways and therefore, it is a multi-media pollutant [8]. The major source of lead in the body is atmospheric (dust) [9,10,11,12,13], polluted soil [14,15,16], contaminated water [15,17,18], food [19], and general urban conditions [20]. Given the differences between environment of individuals at work and at home, lifestyle and socioeconomic status, the specific sources of lead exposure vary widely. The most significant factors that give rise to lead contamination of urban environments and excessive levels of blood lead in urban population is the anthropogenic releases of lead by industrial and transportation emissions [21].

**Figure 1:**
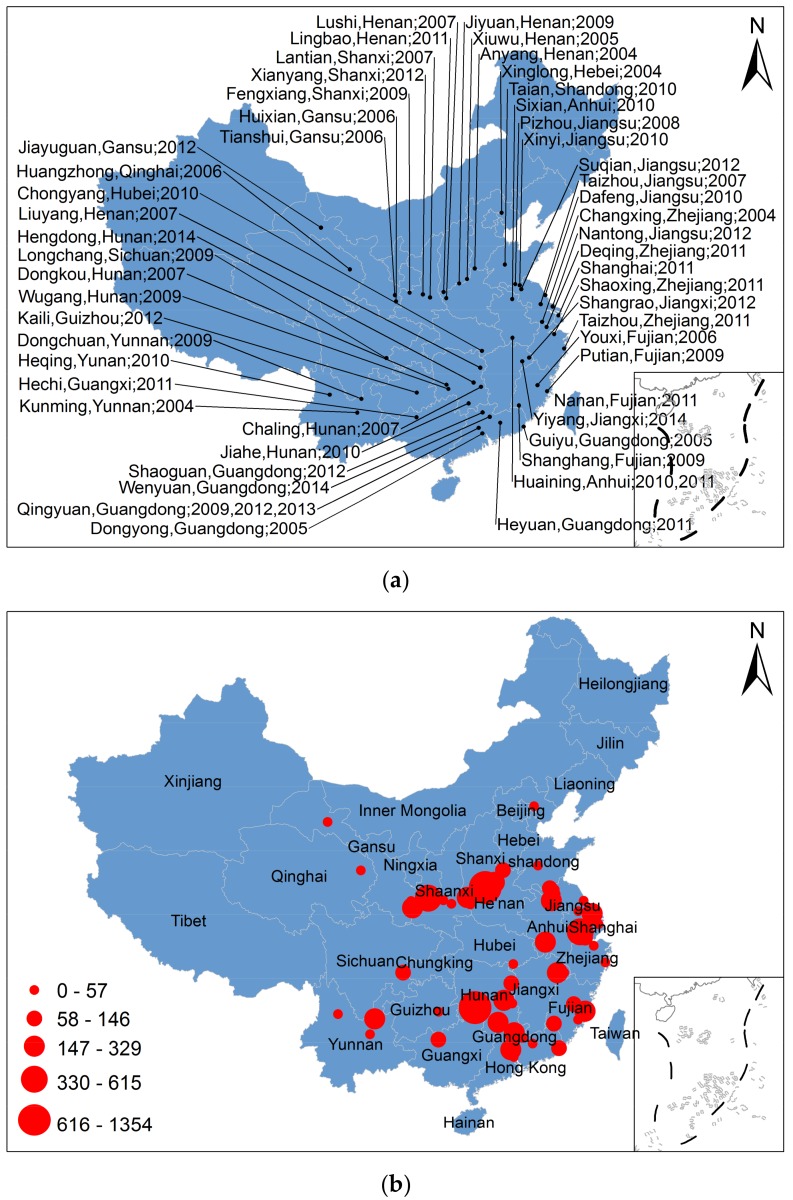
Lead poisoning cases (**a**) and the number of children poisoned in each event, China (**b**).

Since the revelations about the elevation of children’s blood lead (CBL) and health effects of exposure in the 1970s, reducing lead pollution became a major concern. The outcome was efforts to limit lead in paint and ban lead additives in petrol in the Western World. China completely banned the production, sale, and use of leaded petrol in 2000, but lead poisoning incidents continue to frequently occur [22,23,24]. For examples, in August, 2009, 615 children in Fengxiang County, Shaanxi Province, had blood lead levels higher than acceptable level in China (100 μg/L) and at least 166 of the children had blood lead levels higher than 250 μg/L and were sent to a hospital for treatment [25]. The source of pollution was the discharges from a local smelter. During 1 January 2004 to 30 December 2014, lead poisoning in several provinces of China affected more than 10,747 humans and 8091 children (Figure 1) [26]. Most incidents occurred in eastern and central China, where lead smelters and battery enterprises were the main sources of pollution. In recent years, with the development of the Northwest’s economy, many manufacturing industries began operation thereby sharply increasing lead emissions [27].

### 1.2. Overview of Children’s Environmental Lead Exposure

Lead is a non-degradable element which can enter the body through food, water, air, and soil. Lead exposure interferes with the body’s normal physiological and biochemical reactions resulting in damage to the body’s nervous, cardiovascular, and reproductive system. If a mother is exposed to lead during her childhood the lead follows calcium and is stored in her bones. Then, during pregnancy calcium contaminated with lead passes from the mother’s bones through the placenta and can interfere with fetal development. In this way lead exposure is intergenerational passing from the mother to her embryo and fetus. After birth, infants may continue to be lead exposed from the mother’s breastmilk. During infancy and early childhood, the environment becomes an increasingly important source of lead exposure. 

In general, lead in the environment enters the body via the digestive tract and the respiratory tract [28]. Research indicates that the absorption rate of lead in children’s digestive tract is about 42~53%, which is 5~10 times that of adult. The child’s lead excreting capacity is only 65% of adults. Potential lead exposure sources are drinking water, daily diet, toys and stationery, home environment, automobile exhaust, factory pollution, and play areas [29,30]. Instinctively, children put their hands, toys and other things into their mouths. Through these hand-to-mouth behaviors, lead in the environment easily enters the child’s body [31].

In recent years, there has been a remarkable decline in blood lead levels among children [32], especially after the prohibition of the use of leaded gasoline and reduction of lead aerosols in China [33,34]. This situation is related to China’s policy for regulatory control and prevention of lead. China issued a series of regulations to strengthen the management of lead including the national environmental and health action plan, the lead-zinc industry access plan, the 12th Five-Year plan for heavy metal pollution prevention and control, and a series of specific actions to regulate the lead-acid battery industry. The implementation of these policies and regulations has played a major role in restraining industrial lead pollution. At the same time, due to massive blood lead exposure events, people’s attention has continued because, in China large numbers of children continue to be exposed to unacceptable levels of lead.

### 1.3. Toxicity of Lead

The toxic nature of lead has been well known since 2000 BC. Lead is a poison of multiple organ systems including the peripheral and central nervous systems and kidneys [35]. Children, especially those under six years old, are deemed to be the population with highest risk of lead poisoning [36]. Children are more vulnerable and susceptible to lead than adults [37]. High levels of exposure to Pb causes irreversible health effects on children’s neurological and cognitive abilities [38]. Recently, many studies have confirmed that even low levels of lead exposure have adverse effects on children’s intelligence, cognitive ability, and behavior [39,40,41,42]. There is no known safe level of lead exposure.

China is the world’s most populous country, with about 120 million children aged between 0 and 6 years old, accounting for 9.6 percent of the population [43]. Due to the unique characteristics of the valley cities in NW China [44], children living in valley cities are at a higher risk of lead poisoning than children living in other urban areas. There are few studies on environmental pollution in valley cities in NW China, and the health effects of children’s lead exposure are not known. Therefore, the objective of the present study is to study the environmental characteristics and family perceptions about children’s lead exposure in the valley cities of China. The survey about the sources of lead pollution and parental perception of the behavior of children in NVC provides the basis for subsequent research.

In Northwestern China owing to the subjective bias of parents to CBL, it is difficult to collect blood samples from children. Parents believe their children are so healthy that their blood will not require screening. The survey is an alternative way to study lead exposure. A survey gathers information and at the same time communicates knowledge about lead exposure and CBL to the parents. Through questionnaires, high lead areas and communities can be targeted that may also have high CBL. A survey can be used for assessment and then followed up with in-depth study, including tracking air lead, dust, soil, and water in the home and outdoor environments. In the current research, a survey is administered to determine possible exposure to lead by children (<6 years) in terms of environmental characteristics and family factors. Given the difficulties with collecting children’s blood, it is a practical way to evaluate the status of lead exposure of children in Urumqi, Lanzhou, Xining and Yan’an, Northwestern Valley Cities in China.

## 2. Materials and Methods

### 2.1. The Study Area Profiles

Valley cities are widely distributed in the mountainous and hilly terrain of Northwestern China. Northwestern Valley Cities (NVC) have a topographical characteristic that affects the quality of local urban environments [45]. The topography of NVC is closed, especially in winter when weather inversions occurs, resulting in the concentration of severe air pollution. Urumqi, Xining [46], Yan‘an, and Lanzhou are important industrial NVC for mineral energy development. Generally, the environmental atmosphere, water, and land pollution in those cities is more concentrated than the counterpart cities elsewhere, and it is a prominent issue for China [47]. Despite the dramatic increases in economic development of western regions, a large gap exists between these regions and eastern coastal regions. For example, medical facilities in the western region have not kept pace with economic development, and the extent of children’s blood lead exposure is not well understood [48].

### 2.2. Investigation Method

**Figure 2:**
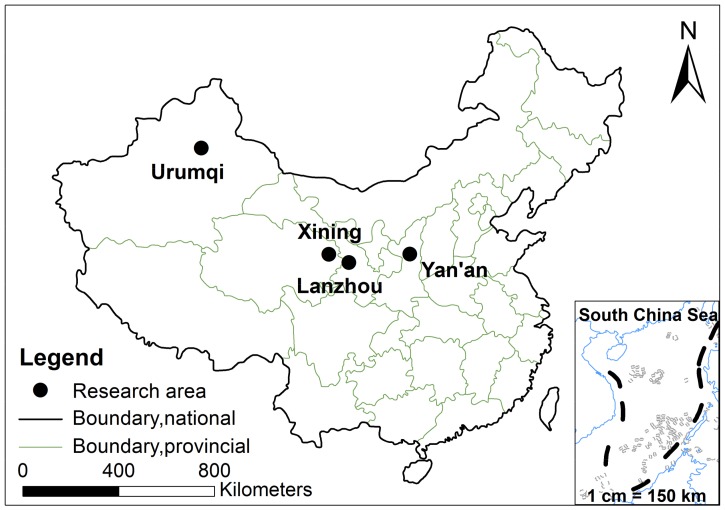
The research area.

The surveys were performed within the legal framework of the China under a project granted by Natural Science Foundation of China, and the questionnaire and field investigation have been already approved by Institutional Research Committee and Review Board. The study interviewed a random sample of 596 families from four typical valley cities in northwestern China (Urumqi, Lanzhou, Xining and Yan’an) (Figure 2). The inclusion criterion was that the families have a <6-year-old child. A sample of 636 families were randomly selected within communities, along streets, from kindergartens, and nearby hospitals.

Forty families were excluded for several reasons (missing important information, or children’s age over 6 years) giving a final total of 596 families included in the study. The questionnaire (Table 1) was administered to eligible families to obtain information on the basic situation of the parents, the mother during pregnancy, and the behavior of children. All survey personnel were trained in administering the questionnaire and recording the information. The statistics were analyzed using SPSS (IBM Corp. Released 2013. IBM SPSS Statistics for Windows, Armonk, NY, USA) and the results were plotted with GIS software (ESRI, 380 New York Street, Redlands, CA, USA). 

### 2.3. Classification of Children’s Symptoms and Behaviors

According to diagnostic criteria for lead poisoning in the “guide for children’s lead poisoning” developed by the Centers for Disease Control and Prevention [49] in the United States and referring to large quantities of literatures, the symptoms would be divided into four levels (in this article, the first level was lowest and the fourth level was highest). When the blood lead level is less than 100 μg/L, fetal toxicity is already apparent. It makes pregnant women abort, preterm, and intrauterine growth retardation, but for children, it belongs to the acceptable level, so we attribute sleeping poorly and biting fingers frequently to the first level. When the level of blood lead is between 100–199 μg/L (level 2), it can affect the nerve conduction velocity and cognitive ability, so that the children are prone to dizziness, irritability, distraction, and hyperactivity.

When children’s blood lead level is at 200–449 μg/L (the third level), children will appear stunted, with low immunity, poor vision, exhibit hearing loss, and slow reactions. In the fourth level, the blood lead level is between 450–699 μg/L, which belongs to severe lead poisoning. Children have symptoms such as big changes in character, learning disabilities, and so on. The relationship between level of blood lead and children’s behavior is shown in Table 2.

## 3. Results and Discussion

### 3.1. Children and CBL

#### 3.1.1. Basic Characteristics of the 596 Children

There were 305 boys, 278 girls and 13 undisclosed gender for the children; mean age of 1.8 ± 1.5, varying from 0 to 6 years and their ages were evenly distributed in infants, early childhood, and preschool. The ethnic groups of the children surveyed were 472 Han Chinese, 74 Hui group, 18 Tibetans, 32 others. Among all children, more than 50% of the children had better physical fitness, 24.7% of the children had a general constitution, 12.2% of the children had poor physique, and 12.2% of the children did not disclose their physical condition (Table 3). All the investigated children were in good physical condition and had no congenital disease.

**Figure 3:**
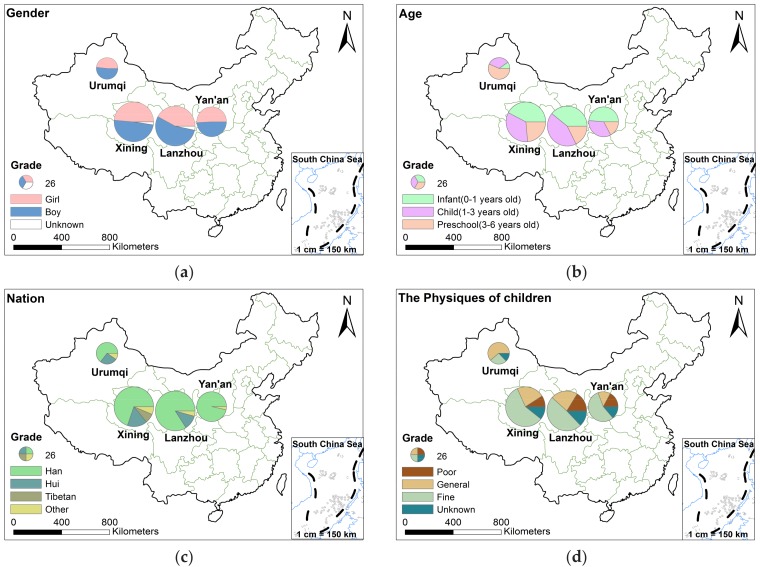
The basic situation of participants. (**a**) for child’s gender; (**b**) for child’s age; (**c**) for ethnic identity (nation); (**d**) for children’s physiques.

Figure 3 below shows the basic characteristics of the 596 children and their parents. The pie size represents the number of people surveyed in each area. Lanzhou and Xining had the largest number of surveyed people and Urumqi the fewest. It was observed that the proportion of girls and boys in all surveyed children were roughly 1:1 (Figure 3a), this was in line with the trend of the total ratio of 51.4:46.4. In Urumqi, the number of children surveyed in different age groups was: preschool > child > infant, and in other three cities, it decreased in the order of infant > child > preschool (Figure 3b). More than 70 percent of the surveyed children were of the Han ethnic group, with a small number of Hui and Tibetan minorities among all participants; more than 20% of the participants were Hui in Xining, Lanzhou and Urumqi (Figure 3c). Although more than 50% of the children’s physical fitness were better, Urumqi still presented about 60% of the children’s physical conditions were in general (Figure 3d). The health of the investigated children in Urumqi were not as good as the other three cities.

From the following Table 4, it was found that with the increase of age, the rate of excessive blood lead was on the rise. The ratio of excessive blood lead in infants were lowest, and were highest in preschools. The growth trend was similar to some developing countries [50,51], while the highest levels of CBL in Europe and America were 1–3 years old [52]. In this study, the effect of gender on CBL was not obvious, and the rate of excessive blood lead in boys were slightly higher than that of girls. In the domestic research, some scholars’ research showed that the blood lead concentration of boys was generally higher than girls [33,53], and another part of scholars showed that there was no significant difference in blood lead level between boys and girls [54,55]. At the same time, the better the children’s physique, the lower the excessive blood lead, the worse the physique, the higher the rate of blood lead.

#### 3.1.2. The Effect of Children’s Behavior on CBL

Referring to a large volume of literature [33,56,57,58,59,60,61], we selected eight children’s behaviors that were relevant to CBL. Table 5 below shows that more than 50% children in four cities had the following habits: eating fruits and vegetables regularly, supplement nutrition, washing their hands regularly, going out for more than 4 h a week, and often contacting lead-containing substances. 11.9% of children liked crawling on the ground, and 21.3% of the children liked licking fingers and eating high lead food. Few children liked crawling on the ground, and a large number of children often washed their hands to show that children had good behavior habits. About 50% of children often eaten fruit/vegetables and given nutritional supplements, which indicated that surveyed children in this four cities had good eating habits, and their parents attached importance to children’s nutritional balance. About 70% of children regularly contacted lead-containing items and went out for more than 4 h a week.

The survey results in Table 6 shows that licking fingers and crawling were having less effect on children’s blood lead level, but many domestic and foreign scholars thought that licking fingers and crawling had a great impact on CBL [62,63]. It might be due to the fact that at least half of the study participants were infants (0–1 year old) who had been under parental care for a long time (for example, washing hand and cleaning frequently), reducing the chances of exposure to dust and soil. However, more than 50% of the children who went out for over 4 h a week appeared symptoms of excessive blood lead, which was because outside activities could be exposed to more dust and soil, increasing the amount of respiratory tract exposure of heavy metal Pb. It was found that long-term contact of lead-containing substances had a significant effect on children’s blood lead level from the Table 6. Although the statistics showed that the proportion of children exposed to lead in the fourth level was lower than other levels, the ratio of all levels was higher than 50%. This might be because gastrointestinal exposure was the main way for children blood lead exposure, and lead-containing items entered children’s body through mouth, resulting in the increase of blood lead in children. Through literature statistics, Horton found that many studies showed metal exposure had an impact on health [64], which result was in line with our study. Therefore, it is very important to guide children to form good diets and behavior habits, which could effectively prevent lead poisoning in children. Schools and families should cooperate effectively to impel children to cultivate good habits.

### 3.2. Parents and CBL

#### 3.2.1. Basic Characteristic of Children’s Parents

Among the parents of all surveyed children, most of them were workers, followed by farmers and civil servants, and many people were not willing to leak their careers (Table 7). Among their fathers, 25.4% had a Master’s or Doctor’s degree, 9.7% had a college degree, 16.1% had a junior college degree, 27.0% had a senior high school degree, and 21.8% did not disclose a degree (Table 7). The proportion of mothers’ education level was basically the same as fathers. At the junior college education level, the number of mothers was higher than that of fathers, and in the senior high school education level, the number of fathers was more than that of mothers.

**Figure 4:**
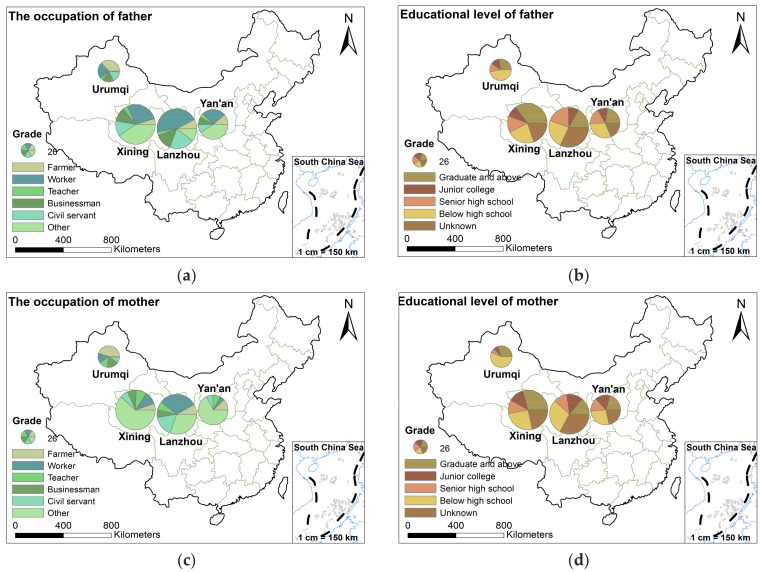
The basic situation of parents (**a**) for father’s occupation; (**b**) for father’s education level; (**c**) for the mother’s occupation; (**d**) for mother’s education level.

The following pictures (Figure 4a–d) illustrate the parents’ occupation and educational level. From Figure 4a,c, we could see that the distribution of parents’ occupation was relatively scattered, especially in Xining and Yan’an, where a lot of parents had not revealed their occupations. In Lanzhou, most of the parents were workers, while in Urumqi, most of the parents were farmers. In Xining and Yan’an, a lot of fathers were workers. The education degree of the participants’ parents in Yan’an had a fairly uniform distribution. In Lanzhou, there were more people who did not disclose their educational background, and the other educational levels were more evenly distributed. In comparison, the number of graduates and above accounted for almost 50% of the number of participants in Xining, much higher than other grades. In Urumqi, below the high school and graduate and above education degree were far more than senior high school and junior college (Figure 4b,d).

Because there were so many types of existing occupations and many people did not disclose their profession, this article has not explored the relationship between the parents’ occupation and CBL. Generally, parents who were highly educated had a high awareness of lead toxicity, which could guide children to avoid relevant risk factors. On the contrary, parents with a low level of education, had less knowledge of lead poisoning, which might increase chance of exposure to lead. The following table (Table 8) shows the relationship between the educational level of parents and CBL. When the education degree of their parents was inconsistent, the highest degree was usually chosen as their final education level. Among the asymptomatic, level 1, and level 2, the largest number of parents had a graduate and above degree. In grade three or four, senior high school and below high school degree had the largest number of parents.

#### 3.2.2. Parents’ Perception of CBL

**Figure 5:**
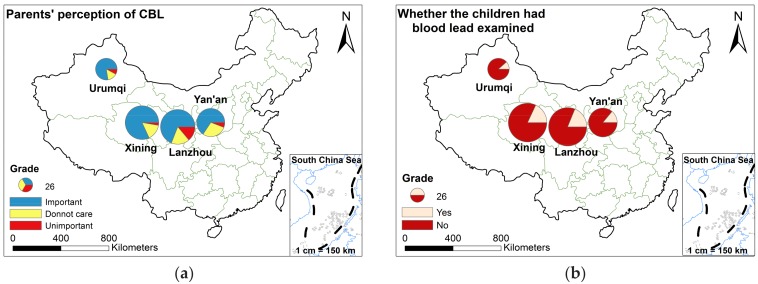
Parents’ perception (**a**) about testing lead in children compared with (**b**) actual blood lead testing in four cities.

Among the 596 participants, 475 parents (80%) expressed their perception about the importance of children’s blood lead (CBL), 73.5% said it was important to check CBL, while 18.7% felt indifferent, and 7.8% thought it was unimportant. The parent’s perception about testing for CBL in four cities was greater than 65% (Table 9). The parents in Xining had the highest attention (82%), for CBL (Table 9, Figure 5a). Among different ethnic groups, most parents perceived that testing for CBL was very important.

Regarding the question of CBL, 554 (93%) responded. Most of the respondents, 83.2% said they had not tested CBL, and only 16.8% had tested blood for lead (Table 9). Statistical analysis of blood lead examination in different ages showed that more than 20% of infants in Lanzhou, children and preschool children in Xining, and preschool children in Yan’an had the blood lead tests already. The number of children who had blood lead test in Xining was more than that in other cities, which was closely related to the parents’ high attention to blood lead. According to the chart below, it was clear that more than 80% of the four cities had not taken the blood lead tests for their children, which was not in accordance with the result of the parents’ attention to the blood lead level in children (Figure 5b). The reason for this phenomenon might be that the medical infrastructure and medical care were inadequate. Although people were aware of the importance of blood lead in children, they were still slightly slow in action due to short of the medical care and necessary health guidance. Therefore, it was suggested that the government should incorporate CBL testing in routine health screening to raise awareness about blood lead in children.

With the improvement of education degree, parents should pay more attention to children’s blood lead [65]. Among the 470 parents, 380 showed the definite response on blood lead in children, and 73.9% of which thought it was important to check blood lead and lead excretion. More than 80% of parents with college degree and above believed that examination of blood lead and lead excretion was very important to children, the ratio of which was far higher than the average level. Although in this study, parents with below high school education paid more attention to CBL than that of high school educated parents, on the whole, parents hold college degree had the similar degree of concern to children’s blood lead (Table 10). 

By contrast, the parents who were below high school education level thought that CBL was important, which was not consistent with our preconceptions. The reason for this might be that the sample size was uneven. However, many parents ignored the importance of CBL, and seemed to have no idea or care for the CBL, which indicated that those parents did not get the information from children care and/or health education practitioners.

#### 3.2.3. The Effect of Mother’s Behaviors during Pregnancy on CBL

Through the investigation and statistics, we found that about 50% of mothers had supplemented calcium and iron during pregnancy, 59.6% of mothers usually walked on the road more than 30 min a day during pregnancy, 75.2% of mothers often used electronic touchscreen products during pregnancy, 21.6% of mothers liked reading during pregnancy, 9.4% mothers who had eaten preserved eggs during pregnancy (Table 11). There were more mothers who had eaten preserved eggs or read newspapers during pregnancy in Urumqi, and in Lanzhou was relatively small. Proper nutritional supplement during pregnancy not only benefits the body of the fetus and mother, but also effectively inhibits the blood lead concentration in the body. In this survey, more than 75% mothers used electronic touchscreen products frequently. Although electronic products did not necessarily have an impact on blood lead, radiation could cause some harm to the fetus, so this behavior should be generally avoided.

Normally, lead in the body is stored in the bones, and it could be released back into blood. When the elements such as calcium and iron were deficient in the body, the lead in the skeleton could be released into the blood. Due to the relatively serious calcium deficiency in pregnant women, the release of lead would increase, which had a greater impact on the fetus. Therefore, it is important to supply calcium and iron to mothers during pregnancy. From Table 11, we could see that mothers paid more attention to nutritional supplements. Table 12 shows that supplementation of calcium, iron and other nutrients during pregnancy inhibited the increase of blood lead. Of all the children with excessive blood lead, more than 50% of mothers used electronic touchscreen products frequently and walked more than 30 min on the road daily during pregnancy. That was to say, the above two factors had a certain influence on the blood lead in children. Compared with mother’s behavior during pregnancy, it could find although mothers spent more time on outgoings during pregnancy, children’s outdoor activities were more inclined to influence their blood lead levels. The reasons would be attributed to: On the one hand, maternal blood lead could only be delivered to children through intrauterine exposure, but the half-life of lead in the human blood was about one month, so high blood lead levels had a greater impact on infants during the pregnancy. On the other hand, lead accumulated in the atmosphere about 1–1.5 m away from the ground, while 75–100 cm away from the ground was just the breathing zone of the children [66]. In the first, second and third level, it was found that more than 70% mother used touchscreen electronic products during pregnancy, implying that using touchscreen electronic products had a certain impact on CBL. Because the touchscreen electronic products include lead compounds, when children’s mothers touched the screen, the toxic lead could enter the body through her skin and impact on blood lead concentration.

It was found that walking on the road for more than 30 min a day and using touchscreen electronic products had negative effects on CBL, and regular supplement of nutrients could effectively decrease blood lead concentrations. Therefore, the supplementation of maternal nutrition should be strengthened and protective measures should be done when outdoor activities were carried out.

### 3.3. Environment and CBL

#### 3.3.1. The Effect of General Residence Conditions on CBL

The lead in the environment comes from industrial emissions, automobile exhaust, coal, ferrous metallurgy, lead containers, toys, family decoration materials, cosmetics and so on. Due to the limitation of the traditional industrial layout, most of industries are concentrated in large densely populated cities, which is one of the main reasons for the increase of CBL.

After investigating the family environment of the participants, we found that about 60% of the families in four cities lived near factories, garages and construction sites, along the downtown or commercial streets. This might be because the four cities are important industrial cities in Northwest, and the main object of the survey is urban children. More than 30% of the participants lived in single floor bungalows, with paint, floor and painted furniture (Table 13).

More than 50% of children with symptoms of excessive blood lead had the following characteristics: their homes were near factories, garages and construction sites around the residential area [67], the distance from downtown or a commercial street was less than 500 m [68,69] (Table 14). Gao [70] and Li [67], in their own studies indicated that the above characteristics were risk factors for elevated blood levels in children. Previous studies have shown that the closer the ground was, the higher the concentration of lead in the atmosphere was. Therefore, children living on lower floors had higher lead exposure than children living on higher floors, which was consistent with the result of the statistics that the higher the level of blood lead, the larger the proportion of children living in a bungalow/on a first floor in the investigation (Table 14). In his study, Chen showed that family residence on a first floor or in a bungalow were the main risk factors for children’s blood lead [71]. The survey showed that there were factories, garage and construction sites around the residential area, and distances from downtown or a commercial street of less than 500 m were associated with CBL. Generally, the concentration of lead in industrial areas is obviously higher than that of ordinary residential areas. Lead pollution around industrial areas is serious, and the risk of lead exposure to children living around industrial areas is higher. Downtowns and business districts are important transport arteries, and lead could enter the environment through the dust and automobile exhaust, causing environmental pollution [58]. By investigating the risk factors of childhood lead poisoning among children aged 0–6 years old in China, Fan concluded that the level of CBL near main roads was higher [59]. In the third and fourth level, the probability of using paint in the home was much higher than that in the low grade (Table 14), and the main reason for this situation might be that most of the decoration materials containing heavy metal lead, which leaded to the increase of lead exposure to children.

Thus, CBL was closely related to the living environment. While cultivating good habits of children, we should also create a good family environment and social environment. The state should formulate relevant policies and strengthen supervision to effectively reduce the level of environmental lead, and parents should try their best to reduce lead exposure in their homes.

#### 3.3.2. The Blood Lead Level of Children in Different Areas

**Figure 6:**
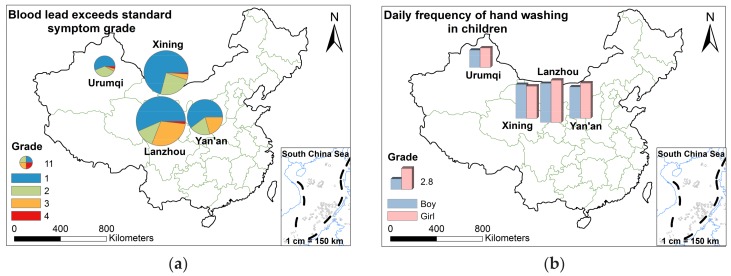

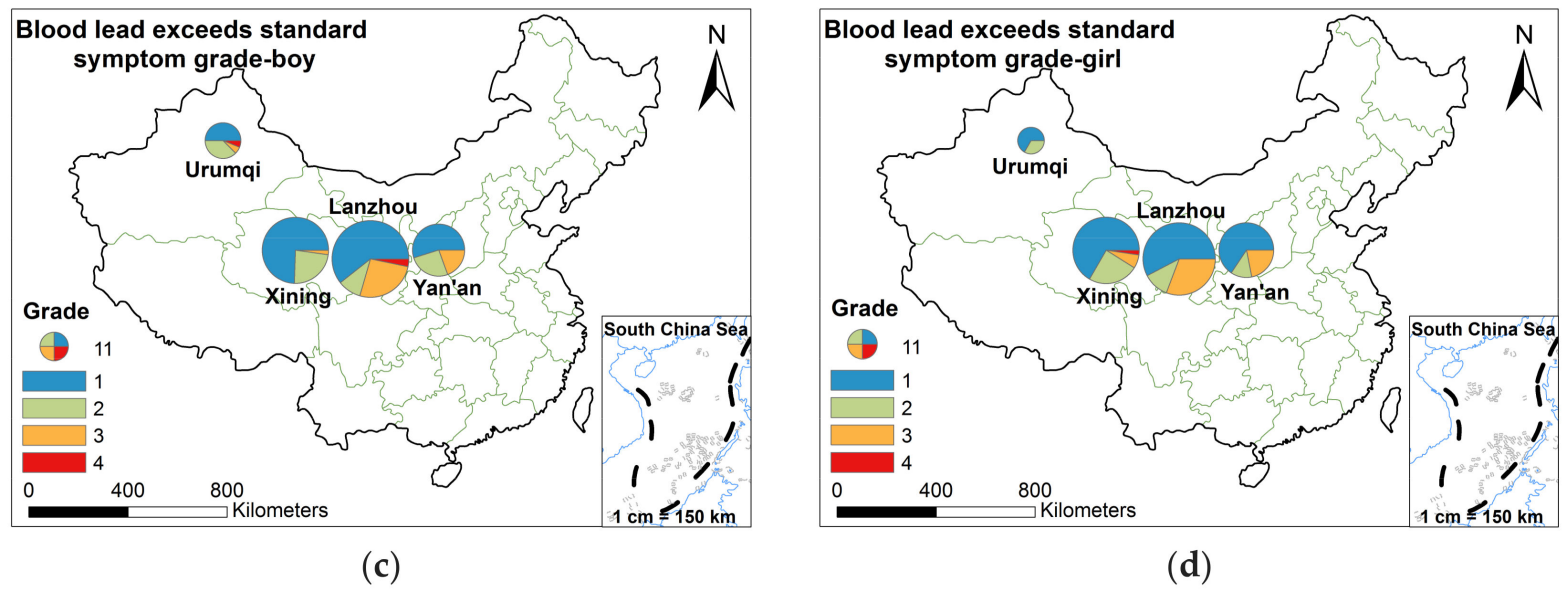
Frequency of hand washing and symptoms of excessive blood lead in children in four cities (**a**) for excessive CBL level symptom; (**b**) for the frequency of hand washing; (**c**) for excessive CBL level symptom in boys; (**d**) for excessive CBL level symptom in girls.

As can be seen in Table 15, more than 40% of respondents indicated that they perceived their children showed symptoms of excessive lead in the blood. Especially in Lanzhou and Yan’an, the situation was more serious, and the rate of the perceived symptoms for CBL was up to 55%. This implied that many children in the four Northwestern Valley cities, might already had high blood exposure. Therefore, it was important for parents and children to be aware of preventive health education. In the illustration below, parents who perceived no symptoms of excessive blood lead were excluded, and it included only parents who perceived with symptoms of excessive blood lead. After eliminating children with no symptoms of blood lead, the statistics still showed that the number of children with perceived blood lead in Lanzhou and Xining were much larger than in Urumqi and Yan’an, which was the same as the original investigation. Figure 6a shows that the level 1 was much higher than other grades in four cities. In Xining and Urumqi, more than 90% of children showed symptoms of excessive blood lead level 1 or level 2, and a few children had level 3 or level 4 symptoms (Figure 6a). This was probably because more than 75% of parents in Urumqi and Xining believed that children’s blood lead was vital, that’s to say, their parents attached much importance to children’s blood lead, so in these two cities, though children appeared to have the symptoms of excessive blood lead, most of symptoms were mild lead poisoning. More than 20% of children in Lanzhou and Yan’an had the third level symptoms, especially in Lanzhou. This might be because Lanzhou and Yan’an are the main mineral energy cities in the Northwest, and lead emissions and pollution are highest in these cities.

To explore the relationship between the children’s daily behavior, for example, frequency of handwashing and the symptoms of excessive blood lead in different gender results. The frequency of handwashing of boys and girls, respectively, and the number of children who showed different blood lead symptoms were investigated in detail and plotted in Table 15. As speculated, the frequency of handwashing per day of boys was lower than that of girls. Among the four cities surveyed, in Xining was the frequency of daily handwashing of boys was higher than that of girls (Table 15, Figure 6b). By comparing the symptom maps of blood lead excess, we found that there were more girls with higher number of blood lead symptoms than boys in Xining, which might be due to the higher frequency of handwashing for boys (Figure 6c,d). Frequency of handwashing was consistent for children in the other cities, so it appeared that handwashing might eliminate the digestive component of lead dust contamination [72]. In China, a lot of researchers have arrived at the same conclusion [73,74].

### 3.4. Prevention of Lead Poisoning in Children: China Is in Action

At present, China has made great efforts in the prevention and control of children’s blood lead, especially in environmental construction. In order to build a beautiful China, China have added the construction of an ecological civilization to the general layout of construction of socialism with Chinese characteristics and build a healthy China into a national strategy. At the same time, China attaches great importance to, and vigorously promotes ecological culture education, so as to enhance people’s awareness of the need for environmental protection.

In addition, in order to curb environmental pollution, China has formulated a series of laws and policies, such as, elimination of leaded gasoline; the transformation of coal fuel to diesel, natural gas and other clean energy sources, and has achieved remarkable results. Although remarkable progress has been made in building an ecological civilization, there are still many policies and measures that cannot be implemented in some of locations, such as NVC. Therefore, local regulatory policies need to be formulated to reduce lead emissions from many local lead-related industries and increase renewable energy development in regional scale, and also, stress the CBL screening in 0–6 years old and children’s health care as well. Meanwhile, the Chinese government had implemented the “egg milk project” in kindergarten and primary school many years ago, strengthening the supplementation of children’s nutrition, and effectively preventing children’s lead poisoning.

## 4. Conclusions

Children’s lead exposure is an exceptionally important health issue. Exposure not only depends on the children’s environment, but also reflects family behavior patterns, and the innate characteristics of children. The study provides supporting statistical evidence that children’s home environment and their behavior, parents’ education level, and mothers’ pregnancy behavior were risk factors of children’s blood lead in Urumqi, Lanzhou, Xining and Yan’an, four Northwestern Valley Cities (NVC) of China. The most striking result of the study was that more than 40% of the children in NVC were perceived to have symptoms of excessive blood lead, but only 16.8% of the children had a blood lead test. The risks of lead exposure were associated with children’s routine handwashing, their outdoor behaviors, and the gestational conditions of their mothers. Although some of parents were aware of the importance of blood lead in children, they were reluctant to act due to lack of the medical care and necessary health guidance. People’s fundamental knowledge about lead hazards and their associated effects on children’s health requires education about prevention. The survey assists with this educational outreach. In addition, government policies that incorporate blood lead testing into routine health screening, raise the health security of children by adding blood lead testing into kindergarten children’s physical examination, and that require blood lead determination as a part of a health examination to monitor the blood lead levels of children are lacking. In addition, realizing that there is no known safe level of exposure for children, and that NVC are particularly vulnerable to environmental contamination, the following policy goals are appropriate: Establish a program and formal procedure to monitor Pb in air, water, and soil; develop health-based standards for Pb in air, water, and soil; create a national clean air, water, and soil program aimed specifically at preventing childhood lead exposure. The benefits of prevention far outweigh the lifelong social and health costs associated with childhood lead exposure.

### Limitations

A limitation of this study is that parent’s perception of various lead exposure symptom levels, as assigned by the CDC (Table 2), are used as a surrogate for actual children’s blood lead measurements. Although lead exposure is generally considered asymptomatic, there are behavioral characteristics, as noted in Table 5, that are associated various CBLs. In this study only 16.8% of the respondents had undergone measurements of CBL. If a multi-factored questionnaire could be used instead of children’s blood lead that would be a breakthrough given the limited access to children’s blood lead. Follow-up study is needed that includes the collection of children’s blood samples to measure CBL and verify if parental perceptions match the multi-factored questionnaire for predicting the CBL in China.

## Figures and Tables

**Table 1:** Lead exposure sources and behaviors of children in 4 Northwestern Valley Cities, China.

General residence conditions	Bungalows or the first floor Whether there located factories, garages, construction sites Degree of decoration (Whether to use paint, floor and paint furniture) Distance from downtown or commercial street <500 m	* Related question, please see the details in Questionnaire Sheet of Question 1 (Q1), Appendix A.
Parents situation	The father’s degree of education The mother’s degree of education Whether the parents have occupational history of exposure to lead	* Related question, please see the details in Questionnaire Sheet of Q2, Appendix A.
The situation of the mother during pregnancy	The main diet With or without iron, calcium supplement With or without preserved egg Walking on the road for more than 30 min each day	* Related question, please see the details in Questionnaire Sheet of Q3, Appendix A.
The basic situation of the child	Child’s gender and age Height and weight Ethnic and physical fitness Whether to eat high lead food(potato chips, popcorn, fried dough sticks, preserved egg, canned food or canned drinks) Whether fruits and vegetables were consumed Contact leaded items Colored plastic products Pencils, coloring books, newspapers, magazines Colored toys, color rubber cement, etc. Colored furniture and colored tableware Color printing food packaging Battery Scented wax products Electronic screen, such as LCD TVs, mobile phones, computers Lipstick and other cosmetics Paints articles Weekly outdoor activity time >4 h Whether the child washes hands, how many times a day Whether the child likes licking his/her fingers Whether the child likes crawling on the ground Whether nutritional supplements were given (vitamin, calcium, cod liver oil)	* Related question, please see the details in Questionnaire Sheet of Q4, Q5 and Q6 Appendix A.
Whether the child has symptoms of blood lead level exceeding	Dizziness, nausea, muscle weakness, fatigue Picky eaters, anorexia, hyperactivity, inattention Capricious, impulsive, irritable, grumpy Stunting such as height, language, hearing and other levels bellowing their peers Immunocompromised, often got cold fever Learning disabilities, and reading ability decreased Biting finger frequently Poor sleep	* Related question, please see the details in Questionnaire Sheet of Q4, Appendix A.
Blood lead measurement	The children had or did not have blood lead inspection Consider it important Consider it does not matter Consider it not important	* Related question, please see the details in Questionnaire Sheet of Q2, Appendix A.

* note: The last list is the corresponding question mark of the questionnaire.

**Table 2:** The relationship between children’s behaviors and the level of blood lead.

Level (i.e., Grade)	Children Blood Lead Level	Standards on Children Lead Poisoning (CDC) [49]	Behavior of Children in Questionnaire
Level 1	<100 μg/L	Relative safety	Biting finger frequently;
			Poor sleep
Level 2	100–199 μg/L	Mild lead poisoning	Picky eaters, anorexia, hyperactivity, inattention;
			Capricious, impulsive, irritable, grumpy;
			Dizziness, nausea, muscle weakness, fatigue
Level 3	200–449 μg/L	Moderate lead poisoning	Immunocompromised, often got cold or fever;
			Stunting such as height, language, hearing and bullying their peers
Level 4	450–699 μg/L	Severe lead poisoning	Severe learning disabilities, and decreased reading ability

**Table 3:** The basic situation of children.

Gender	Proportion	Age	Proportion	Nation	Proportion	The Physiques of Children	Proportion
Boy	51.4	Infant (0–1 year old)	39.1	Han	79.2	Poor	12.2
Girl	46.4	Child (1–3 years old)	37.2	Hui	12.4	General	24.7
Unknown	2.2	Preschool (3–6 years old)	23.7	Zang	3	Fine	50.9
				Other	5.4	Unknown	12.2

**Table 4:** The relationship between the basic characteristics of children and CBL.

Blood Lead Exceeds Standard Symptom Levels	Age			Gender		The Physiques of Children		
	Infant	Child	Preschool	Girl	Boy	Poor	General	Fine
1	94 (40.3)	61 (27.5)	30 (21.3)	87 (31.3)	97 (31.8)	13 (17.8)	39 (26.5)	113 (37.3)
2	5 (2.1)	25 (11.3)	27 (19.1)	24 (8.6)	31 (10.2)	10 (13.7)	15 (10.2)	17 (5.6)
3	10 (4.3)	27 (12.1)	14 (9.9)	26 (9.4)	24 (7.9)	26 (35.6)	7 (4.8)	7 (2.3)
4	N.D.	N.D.	4 (2.9)	1 (0.4)	3 (1.0)	1 (1.4)	2 (1.4)	1 (0.3)
asymptomatic	124 (53.3)	109 (49.1)	66 (46.8)	140 (50.3)	150 (49.1)	23 (31.5)	84 (57.1)	165 (54.5)

N.D. means no data, (note: blood lead exceeds standard symptom level is taken from Table 2).

**Table 5:** Children’s behavior in different cities.

		Lanzhou	Urumqi	Xining	Yan’an	Sum
		(*n* = 209)	(*n* = 62)	(*n* = 208)	(*n* = 117)	(*n* = 596)
Childrens‘behavior	Like crawling on the ground	16 (7.7)	9 (14.5)	29 (13.9)	17 (14.5)	71 (11.9)
	Like licking fingers	53 (25.4)	7 (11.3)	40 (19.2)	27 (23.1)	127 (21.3)
	often eat high lead food	35 (16.7)	40 (64.5)	44 (21.2)	29 (24.8)	148 (24.8)
	Often eat fruit and vegetables	111 (53.1)	31 (50.0)	94 (45.2)	52 (44.4)	288 (48.3)
	Add nutritional supplements	144 (68.9)	28 (45.2)	112 (53.8)	52 (44.4)	336 (56.4)
	Wash your hands regularly	146 (69.9)	41 (66.1)	96 (46.2)	81 (69.2)	364 (61.1)
	Weekly outdoor activity time >4 h	131 (62.7)	39 (62.9)	145 (69.7)	95 (81.2)	410 (68.8)
	Regular contact leaded items	158 (75.6)	56 (90.3)	149 (71.6)	87 (74.4)	450 (75.5)

**Table 6:** The relationship between children’s behaviors and CBL.

		1 (185)	2 (57)	3 (51)	4 (4)
Childrens‘ behavior	Like crawling on the ground	29 (15.7)	4 (7.0)	10 (19.6)	N.D.
	Like licking fingers	68 (36.8)	5 (8.8)	10 (19.6)	1 (25.0)
	Often eat high lead food	31 (16.8)	28 (49.1)	18 (35.3)	2 (50.0)
	Weekly outdoor activity time >4 h	132 (71.4)	46 (80.7)	44 (86.3)	2 (50.0)
	Regular contact leaded items	154 (83.2)	52 (91.2)	41 (80.4)	2 (50.0)

N.D. means no data, (note: blood lead exceeds standard symptom level taken from Table 2).

**Table 7:** The basic situation of parents.

The Basic Situation of the Father				The Basic Situation of the Mother			
Occupation	Proportion	Educational Level	Proportion	Occupation	Proportion	Educational Level	Proportion
farmer	10.4	graduate and above	25.4	farmer	11.7	graduate and above	22.3
worker	32.9	junior college	9.7	worker	17.3	junior college	12.9
teacher	3.7	senior high school	16.1	teacher	8.4	senior high school	11.3
businessman	12.2	below high school	27.0	businessman	6.5	below high school	30.2
civil servant	14.8	unknown	21.8	civil servant	10.4	unknown	23.3
other	26			other	45.7		

**Table 8:** The relationship between educational level of parents and CBL.

Blood Lead Exceeds Standard Symptom Level (i.e., Grade)	Graduate and above	Junior College	Senior High School	Below High School
1	56 (41.5)	12 (8.9)	26 (19.2)	41 (30.4)
2	20 (40.8)	5 (10.2)	8 (16.3)	16 (32.7)
3	6 (15.4)	4 (10.2)	12 (30.8)	17 (43.6)
4	N.D.	N.D.	1 (50.0)	1 (50.0)
asymptomatic	89 (36.3)	39 (15.9)	49 (20.0)	68 (27.8)

N.D. means no data. (note: blood lead exceeds standard symptom level taken from Table 2).

**Table 9:** Parents’ perception of blood lead in children and blood lead test in four cities.

	Lanzhou (*n* = 158)	Urumqi (*n* = 62)	Xining (*n* = 150)	Yan’an (*n* = 105)	Sum (*n* = 475)
**Parents** **’** **perception of blood lead in children (*n* (%))**					
Important	109 (69.0)	48 (77.4)	123 (82.0)	69 (65.7)	349 (73.5)
Unimportant	22 (13.9)	5 (8.1)	4 (2.7)	6 (5.7)	37 (7.8)
Don’t care	27 (17.1)	9 (14.5)	23 (15.3)	30 (28.6)	89 (18.7)
	**Lanzhou (*n* = 191)**	**Urumqi (*n* = 61)**	**Xining (*n* = 194)**	**Yan** **’** **an (*n* = 108)**	**Sum (*n* = 554)**
**Whether the children had blood lead examined (*n* (%))**					
Yes	36 (18.8)	7 (11.5)	35 (18.0)	15 (13.9)	93 (16.8)
No	155 (81.2)	54 (88.5)	159 (82.0)	93 (86.1)	461 (83.2)

**Table 10:** The relationship between educational level of parents and the perception of the importance of blood lead (*n* (%)).

Educational Level of Parents	Important	Unimportant	Don’t Care
Graduate and above	108 (80.0)	9 (6.7)	18 (13.3)
Junior college	35 (71.4)	2 (4.1)	12 (24.5)
Senior high school	53 (69.7)	3 (3.9)	20 (26.4)
Below high school	85 (70.8)	14 (11.7)	21 (17.5)
Sum	281 (73.9)	28 (7.4)	71 (18.7)

**Table 11:** Mothers’ behavior during pregnancy in different cities.

		Lanzhou	Urumqi	Xining	Yan’an	Sum
		(*n* = 209)	(*n* = 62)	(*n* = 208)	(*n* = 117)	(*n* = 596)
Mother’s behavior during pregnancy	Taking calcium supplements	69 (33.0)	35 (56.5)	121 (58.2)	80 (68.4)	305 (51.2)
	Taking Iron supplements	131 (62.7)	34 (54.8)	84 (40.4)	34 (40.4)	283 (47.5)
	Eating preserved eggs	18 (8.6)	18 (29.0)	11 (5.3)	9 (7.7)	56 (9.4)
	Walking on the road for more than 30 min each day	128 (61.2)	36 (58.1)	116 (55.8)	75 (64.1)	355 (59.6)
	Using touchscreen electronic products	158 (75.6)	42 (67.7)	153 (73.6)	95 (81.2)	448 (75.2)
	Reading newspaper	41 (19.6)	27 (43.5)	49 (23.6)	12 (10.3)	129 (21.6)

**Table 12:** The relationship between mothers’ behavior during pregnancy and CBL.

Blood Lead Exceeds Standard Symptom Level (i.e., Grade)		1 (185)	2 (57)	3 (51)	4 (4)
Mother’s behavior during pregnancy	Taking calcium supplements	97 (52.4)	41 (71.9)	25 (49.0)	2 (50.0)
	Taking Iron supplements	97 (52.4)	28 (49.1)	25 (49.0)	2 (50.0)
	Walking on the road for more than 30 min each day	107 (57.8)	29 (50.9)	39 (76.5)	3 (75.0)
	Using touch screen electronic products	142 (76.8)	45 (78.9)	38 (74.5)	1 (25.0)
	Reading newspaper	51 (27.6)	6 (10.5)	7 (13.7)	N.D.

N.D. means no data. (note: blood lead exceeds standard symptom level taken from Table 2).

**Table 13:** General residence conditions in different cities.

		Lanzhou	Urumqi	Xining	Yan’an	Sum
		(*n* = 209)	(*n* = 62)	(*n* = 208)	(*n* = 117)	(*n* = 596)
General residence conditions	Bungalows or the first floor	75 (35.9)	16 (25.8)	45 (21.6)	43 (36.8)	179 (30.0)
	Located near factories, garages, construction sites	127 (60.8)	45 (72.6)	122 (58.7)	61 (52.1)	355 (59.6)
	Distance from downtown or commercial street <500 m	168 (80.4)	11 (17.7)	128 (61.5)	76 (65.0)	383 (64.3)
	Use paint, floors and painted furniture	58 (27.8)	39 (62.9)	56 (26.9)	39 (33.3)	192 (32.2)

**Table 14:** The relationship between general residence conditions and CBL.

Blood Lead Exceeds Standard Symptom Level (i.e., Grade)		1 (185)	2 (57)	3 (51)	4 (4)
General residence conditions	Bungalows or the first floor	53 (28.5)	17 (25.4)	27 (52.9)	2 (50.0)
	There located factories, garages, construction sites	114 (61.3)	32 (47.8)	37 (72.5)	2 (50.0)
	Distance from downtown or commercial street <500 m	117 (62.9)	36 (53.7)	38 (74.5)	3 (75.0)
	Use paint, floor and paint furniture	55 (29.6)	14 (20.9)	23 (45.1)	2 (50.0)

Note: blood lead exceeds standard symptom level taken from Table 2.

**Table 15:** Frequency of hand washing and symptoms of excessive blood lead in children in four cities.

	Lanzhou	Urumqi	Xining	Yan’an	Sum
	(*n* = 209)	(*n* = 62)	(*n* = 208)	(*n* = 117)	(*n* = 596)
**Daily frequency of hand washing in children (*n*)**					
Boy	5.114	2.25	4.461	4.123	4.41
Girl	5.545	2.533	4.238	4.627	4.55
**Blood lead exceeds CDC symptom levels (i.e., grade) (*n* (%))**					
1	64 (30.6)	14 (22.6)	66 (31.7)	38 (32.5)	182 (30.5)
2	14 (6.7)	9 (14.5)	22 (10.6)	12 (10.3)	57 (9.5)
3	33 (15.8)	1 (1.6)	4 (1.9)	13 (11.0)	51 (8.6)
4	2 (1.0)	1 (1.6)	1 (0.5)	N.D.	4 (0.7)
Sum	113 (54.1)	25 (40.3)	93 (44.7)	63 (53.8)	294 (49.3)
asymptomatic	96 (45.9)	37 (59.7)	115 (55.3)	54 (46.2)	302 (50.7)
	**Lanzhou**	**Urumqi**	**Xining**	**Yan’an**	**Sum**
	**(*n* = 88)**	**(*n* = 30)**	**(*n* = 101)**	**(*n* = 59)**	**(*n* = 278)**
**Blood lead exceeds CDC symptom levels (i.e., grade)-girl (*n* (%))**					
1	30 (34.1)	6 (20.0)	30 (29.7)	21 (35.6)	87 (31.3)
2	6 (6.8)	3 (10.0)	11 (10.9)	4 (6.8)	24 (8.6)
3	16 (18.2)	N.D.	3 (3.0)	7 (11.8)	26 (9.3)
4	N.D.	N.D.	1 (1.0)	N.D.	1 (0.4)
Sum	52 (59.1)	9 (30.0)	45 (44.6)	32 (54.2)	138 (49.6)
asymptomatic	36 (40.9)	21 (70.0)	56 (55.4)	21 (45.8)	140 (50.4)
	**Lanzhou**	**Urumqi**	**Xining**	**Yan’an**	**Sum**
	**(*n* = 114)**	**(*n* = 32)**	**(*n* = 102)**	**(*n* = 57)**	**(*n* = 305)**
**Blood lead exceeds standard symptom levels (i.e., grade)-boy (*n* (%))**					
1	37 (32.5)	8 (25.0)	35 (34.3)	17 (29.8)	97 (31.7)
2	6 (5.3)	6 (18.8)	11 (10.8)	8 (14.1)	31 (10.1)
3	16 (14.0)	1 (3.1)	1 (1.0)	6 (10.5)	24 (7.8)
4	2 (1.7)	1 (3.1)	N.D.	N.D.	3 (1.0)
Sum	61 (53.5)	16 (50.0)	47 (46.1)	31 (54.4)	155 (50.6)
asymptomatic	53 (46.5)	16 (50.0)	55 (53.9)	26 (45.6)	150 (49.4)

N.D. means no data. (note: blood lead exceeds standard symptom level taken from Table 2).

## Appendix A

Questionnaire Sheet on the Environmental Exposure of Toxic Element Lead to Children (<6 Years) in Northwestern Valley Cities, NW China.

### A.1. Family Environment

*( ) Is your home a bungalow?*

*( ) Does your family live in the building?*

 *If so, which floor do you live on? _____*

*( ) Is your home in downtown/ busy commercial street?*

 *If so, how many meters from the busy commercial street? _____*

*( ) Is there a factory around your home? If there is, what kind of factory or enterprise is it? ____How many meters is your home from the enterprise? ____*

*( ) Is there a parking lot around your home?*

*( ) Is there a construction site around your home?*

*( ) Does the father’s occupation involve lead or electronic assembly?*

*( ) Does the mother’s occupation involve lead or electronic assembly?*

*( ) Which is your household fuel?*

*___ Natural gas ___ coal gas ___ induction cooker*

*___ Hearth ___ the others*

*( ) What* *’* *s the area of your family home?*

*___ <50 m^2^ ___ 50–80 m^2^ ___ 80–120 m^2^ ___ >120 m^2^*

*( ) What is the type of your home decoration?*

*___ new house, living for ___ month(s) ___ old house, living for ___ year(s)*

*___ use paint, floor and paint furniture, etc.,*

*___ use high power electrical appliances such as computers, electromagnetic furnaces and so on*

*___ don* *’* *t use paint, floor and paint furniture, etc.,*

*___ don* *’* *t use high power electrical appliances such as computers, electromagnetic furnaces and so on*

*( ) Questions on the cleanliness of your family?*

*Cleaning frequency: ___time(s)/month*

*How much is the amount of dust each time? ___g*

### A.2. Parents’ Situation

***Father*** *: Occupation*

*___ farmer ___ worker ___ teacher ___ businessman*

*___civil servant ___ soldier ___ student*

*Hobby or job*

*___Electrical maintenance or have a history of contact with lead processing*

*___paints/electrical/lead manufacture/pipelines/welding and so on*

*___decorate/reform/dismantle buildings*

*___make ceramics or lead glaze ceramics*

*___generate chemical substances*

*___the maintenance of radiators*

*___house building or maintenance*

 *___battery production or maintenance*

 *___valve and fittings installation*

 *___ smelting or manufacture of heavy metals*

 *___burning wood with lead paint*

 *___car repair shop or waste recycling factory*

 *___reprocessing of furniture*

 *___fishing*

 *___shooting range*

 *___ other*

 *Educational level*

 *___doctor ___master ___graduate*

 *___junior college ___senior high school ___below high school*

***Mother*** *: Occupation*

*___ farmer ___ worker ___ teacher ___ businessman*

*___ civil servant ___ soldier ___ student*

*Hobby or job*

*___Electrical maintenance or have a history of contact with lead processing*

*___paints/electrical/lead manufacture/pipelines/welding and so on*

*___decorate/reform/dismantle buildings*

*___make ceramics or lead glaze ceramics*

*___generate chemical substances*

*___the maintenance of radiators*

*___house building or maintenance*

  *___battery production or maintenance*

  *___valve and fittings installation*

  *___the smelting or manufacture of heavy metals*

  *___burning wood with lead paint*

  *___car repair shop or waste recycle factory*

  *___reprocessing of furniture*

  *___fishing*

  *___ shooting range*

  *___ other*

  *Educational level*

  *___doctor ___master ___graduate*

  *___junior college ___senior high school ___below high school*

*( ) Do parents have a history of hereditary condition?*

 *___ yes, what kind of condition? ___*

 *___ no*

*( ) Do you think it is important to check children’s blood lead and lead excretion?*

 *___important ___ unimportant ___ do not care*

*( ) Have you done a blood lead test for your child?*

 *___yes, What is the value of blood lead? ___*

 *Has the blood lead exceeded the standard? __yes ___no*

 *___no*

### A.3. Mother’s Situation during Pregnancy

*( ) What kind of food is the main food for the mother during pregnancy?*

 *___pregnant milk powder, which brand? ___*

 *___meats, what kind of meat is the main kind of meat? Such as, beef, chicken, mutton, fish ___*

 *___vegetables, what kind of vegetable is the main kind of vegetable? Such as, tomatoes, sweet potatoes ___*

*( ) Did the mother replenish calcium during pregnancy?*

*( ) Did the mother replenish the iron during pregnancy?*

*( ) Did mother smoke during pregnancy?*

*( ) Did mother drink coffee during pregnancy?*

*( ) Did mother drink alcohol during pregnancy?*

*( ) How many times does the mother walk on the road every day?*

*___<20 min ___30 min ___>30 min*

*( ) Did mother eat eggs during pregnancy?*

*( ) Did mother eat preserved eggs during pregnancy?*

*( ) How long does the mother sleep every day during pregnancy?*

*( ) Did mother use touchscreen electronics during pregnancy?*

*( ) Did mother read the newspaper during pregnancy?*

### A.4. The Situation of Children

 *Sex___ Age___ Nation___ Height___ Weight___*

*( ) Physical condition*

 *___ Poor (getting a fever or illness frequently)*

 *___ General*

 *___ Fine*

*( ) Is your child going to kindergarten?*

 *___yes ___no*

*( ) Does your child have the following performance?*

 *___ Dizziness, nausea, muscle weakness, fatigue*

 *___ Picky eaters, anorexia, hyperactivity, inattention*

 *___ Capricious, impulsive, irritable, grumpy*

 *___ Stunting such as height, language, hearing and other levels below their peers*

 *___ Immunocompromised, often got colds or fevers*

 *___ Learning disabilities, and decreased reading ability*

 *___ Biting nails frequently*

 *___ Poor sleep*

*( ) Have your children owned any of the following toys or contacted the following items?*

 *___ Colored plastic products*

 *___ Pencils, coloring books, newspapers, magazines*

 *___ Colored toys, color rubber cement, etc.*

 *___ Colored furniture and colored tableware*

 *___ Color printing food packaging*

 *___ Batteries*

 *___ Scented wax products*

 *___ Electronic screens, such as LCD TVs, mobile phones, computers*

 *___ Lipstick and other cosmetics*

 *___ Painted articles*

*( ) Do your children often eat or like the following foods?*

 *___ Popcorn, potato chips, and other puffed foods*

 *___ Youtiao*

 *___ Preserved egg*

 *___Canned food or canned drinks*

### A.5. Children’s Eating Habits

*( ) milk powder, which brand: ___ How many milliliters of milk powder does the child drink every day?___*

*( ) breast milk, how many milliliters of milk powder does the child drink every day? ___*

*( ) dairy, what kind of dairy? ___ How many milliliters of dairy does the child drink every day? ___*

*( ) How many times a day to feed the baby? ___*

*( )* *Infant Supplementary Food (such as, vegetables, fruits, farina, nuts and so on)*

*What’s the total amount of vegetables and fruits as a supplementary food? ___g*

 *Which brand? ___*

 *What kind of vegetable? ___*

 *What kind of fruit? ___*

 *What’s the total amount of farina as a supplementary food? ___g*

 *Which brand? ___*

 *What kind of farina? ___*

 *What’s the total amount of nuts as a supplementary food? ___g*

 *Which brand? ___*

 *What kind of nut? ___*

*( ) How many milliliters of water does your child drink a day? ___*

 *___Tap water or ___purified water*

*( ) How many milliliters of juice does your child drink a day? ___*

 *___Fresh juice or ___barreled juice*

*( ) Does child have the habit of washing hands?*

 *___yes, how many times a day? ___*

 *___no*

*( ) Does your child like eating sugars and sweet foods?*

*( ) Is your child picky?*

*( ) Does your child like eating meats? What kind of meat is the main kind of meat? Such as, beef, chicken, mutton, fish ___; ___g/day*

*( ) Does your child like eating vegetables? What kind of vegetable is the main kind of vegetable? Such as, tomato, sweet potato___; ___g/day*

*( ) Does your child like eating noodles? Which brand? ___; ___g/day*

*( ) Does your child like eating rice? Which brand? ___; ___g/day*

*( ) Does your child like eating porridge? Millet porridge ( ) or congee ( ); ___g/day*

*( ) Does your child like licking fingers? How many times a day? ___*

*( ) Do you use pottery, ceramics, or crystal products to cook, eat and drink?*

*___yes; which kind of appliance do you use? ___pottery, ___ ceramics, ___ crystal products*

*___no*

### A.6. Children’s Daily Activity

*( ) Indoor activities: ___hour(s); outdoor activities: ___hour(s), ___times/month*

*( ) Outdoor activity location: ___ square, ___park, ___kindergarten___ other places*

*( ) Does your child like to play with toys?*

 *What kind of material of the toy? ___plastic, ___metal, ___toy with paint on the surface*

 *How long does your child play the toy every day? ___min(s)/day*

*( ) Does your child like to crawl?*

*How many times does your child crawl on the ground every day? And about how long.___, ___*

*( ) Does your child like drawing? Which brushes are used?*

*___pencil ___water color brush ___crayon ___the others*

 *How long does your child draw every day? ___min(s)/day*

*( ) How many times does your child take a bath each month? ___time(s)/month*

*( ) How many times does your child swim each month? ___time(s)/month*

*( ) what* *’* *s the frequency of your child* *’* *s breathing? ___times/min*

*( ) What kind of nutritional supplement do you give your child?*

 *___ Vitamin sugar, ___tablet(s)/day, ___time(s)/day; which brand? ___*

 *___ Calcium tablet, ___tablet(s)/day, ___time(s)/day; which brand? ___*

 *___ Cod liver oil, ___tablet(s)/day, ___time(s)/day; which brand? ___*

 *___ Other nutritional tablets, Which kind? ___; ___tablet(s)/day, ___time(s)/day; which brand? ___*

### A.7. Would You Like to Cooperate with Us to Collect Blood Lead Data for Your Children?

*( ) yes, Please leave your address: ______________________________________*

   *your number:______________________________________*

   *your signature:_____________________________________*

*( ) no*

*( ) yes, but specific consultations are required. Please leave your number: _____*_
